# Molecular and phenotypic profiling of colorectal cancer patients in West Africa reveals biological insights

**DOI:** 10.1038/s41467-021-27106-w

**Published:** 2021-11-24

**Authors:** Olusegun Isaac Alatise, Gregory C. Knapp, Avinash Sharma, Walid K. Chatila, Olukayode A. Arowolo, Olalekan Olasehinde, Olusola C. Famurewa, Adeleye D. Omisore, Akinwumi O. Komolafe, Olaejinrinde O. Olaofe, Aba I. Katung, David E. Ibikunle, Adedeji A. Egberongbe, Samuel A. Olatoke, Sulaiman O. Agodirin, Olusola A. Adesiyun, Ademola Adeyeye, Oladapo A. Kolawole, Akinwumi O. Olakanmi, Kanika Arora, Jeremy Constable, Ronak Shah, Azfar Basunia, Brooke Sylvester, Chao Wu, Martin R. Weiser, Ken Seier, Mithat Gonen, Zsofia K. Stadler, Yelena Kemel, Efsevia Vakiani, Michael F. Berger, Timothy A. Chan, David B. Solit, Jinru Shia, Francisco Sanchez-Vega, Nikolaus Schultz, Murray Brennan, J. Joshua Smith, T. Peter Kingham

**Affiliations:** 1 Faculty of Clinical Sciences, College of Health Sciences, Obafemi Awolowo University, Ile-Ife, Nigeria; 2 Department of Surgery, Memorial Sloan Kettering Cancer Center, New York, NY USA; 3 Marie-Jose and Henry R. Kravis Center for Molecular Oncology, Memorial Sloan Kettering Cancer Center, New York, NY USA; 4 Human Oncology and Pathogenesis Program, Memorial Sloan Kettering Cancer Center, New York, NY USA; 5 Tri-Institutional Program in Computational Biology and Medicine, Weill Cornell Medical College, New York, NY USA; 6 Federal Medical Centre, Owo, Ondo State Nigeria; 7 Department of Surgery, University of Ilorin Teaching Hospital, Ilorin, Nigeria; 8 Department of Surgery, Ladoke Akintola University of Technology, Ogbomoso, Oyo State Nigeria; 9 Department of Surgery, University of Medical Sciences, Ondo, Ondo State Nigeria; 10 Colorectal Service, Department of Surgery, Memorial Sloan Kettering Cancer Center, New York, NY USA; 11 Department of Epidemiology and Biostatistics, Memorial Sloan Kettering Cancer Center, New York, NY USA; 12 Clinical Genetics Service and the Cancer Biology and Genetics Program, Memorial Sloan Kettering Cancer Center, New York, NY USA; 13 Gastrointestinal Oncology Service, Department of Medicine, Memorial Sloan Kettering Cancer Center, New York, NY USA; 14 Niehaus Center for Inherited Cancer Genomics, Memorial Sloan Kettering Cancer Center, New York, NY USA; 15 Department of Radiation Oncology, Memorial Sloan Kettering Cancer Center, New York, NY USA; 16 Department of Pathology, Memorial Sloan Kettering Cancer Center, New York, NY USA; 17 Bobst International Center, Memorial Sloan Kettering Cancer Center, New York, NY USA; 18 Hepatopancreatobiliary Service, Department of Surgery, Memorial Sloan Kettering Cancer Center, New York, NY USA; 19 Present Address: Division of General Surgery, Department of Surgery, Dalhousie University, Halifax, NS Canada

**Keywords:** Cancer genetics, Cancer genetics, Colorectal cancer

## Abstract

Understanding the molecular and phenotypic profile of colorectal cancer (CRC) in West Africa is vital to addressing the regions rising burden of disease. Tissue from unselected Nigerian patients was analyzed with a multigene, next-generation sequencing assay. The rate of microsatellite instability is significantly higher among Nigerian CRC patients (28.1%) than patients from The Cancer Genome Atlas (TCGA, 14.2%) and Memorial Sloan Kettering Cancer Center (MSKCC, 8.5%, *P* < 0.001). In microsatellite-stable cases, tumors from Nigerian patients are less likely to have *APC* mutations (39.1% vs. 76.0% MSKCC *P* < 0.001) and WNT pathway alterations (47.8% vs. 81.9% MSKCC, *P* < 0.001); whereas RAS pathway alteration is more prevalent (76.1% vs. 59.6%, *P* = 0.03). Nigerian CRC patients are also younger and more likely to present with rectal disease (50.8% vs. 33.7% MSKCC, *P* < 0.001). The findings suggest a unique biology of CRC in Nigeria, which emphasizes the need for regional data to guide diagnostic and treatment approaches for patients in West Africa.

## Abstract (web summary)

Understanding the molecular and phenotypic profile of colorectal cancer (CRC) in West Africa is important for early detection and treatment. Here, the authors use a multigene next-generation sequencing panel to identify genomic differences in Nigerian CRCs compared to those from TCGA and MSKCC cohorts.

## Introduction

Colorectal cancer (CRC) is the third most common cancer and the second leading cause of cancer-associated death globally^1^. The number of new cases is predicted to increase by 77% between 2012–2030, and the majority of that growth is projected to occur in low- and -middle income countries (LMICs)^2^. In sub-Saharan Africa, the incidence of CRC is increasing, yet the disease remains poorly characterized in this region^3–5^.

Retrospective series suggest that CRC in Nigeria is phenotypically characterized by a younger age of onset, a higher rate of right colon and rectal primary tumors, and an increased prevalence of mucinous differentiation as compared to American and European populations^5–10^. The molecular profile of CRC in Nigerian patients is poorly characterized, creating an information gap that is increasingly relevant to both prognosis and treatment selection^11,12^. In two small studies at individual tertiary care facilities in Southwest Nigeria, 43.0 and 34.5% of formalin-fixed paraffin-embedded (FFPE) CRC specimens demonstrated microsatellite instability (MSI), respectively^9,13^. A smaller analysis from another single institution demonstrated that 23.0% of CRC specimens exhibited deficient mismatch repair (MMR) protein expression^14^. Evidence also suggests a higher incidence of wild-type *BRAF* (95.5%) and *KRAS* (79.0%) in Nigerian CRC compared to cohorts of primarily Caucasian patients from high-income countries (HICs)^15^. However, these previous studies rely on FFPE specimens that can be a challenge to examine with immunohistochemistry (IHC) in low-resource environments. As a result, existing studies often struggle to evaluate the majority of their patients with MSI testing. In addition, previous studies are single institution with limited retrospective clinical data and no long-term prospective oncologic outcomes.

In this work, we compare the molecular and clinicopathologic profile of CRC in Nigeria to a reference population from HICs. We provide insights into the poorly characterized molecular changes and genomic alterations associated with CRC in West Africa that will facilitate the adoption of evidence-based best pratices and policies to address the rising burden of CRC in the region.

## Results

### Clinical comparison

From Nigeria, 380 patients were enrolled, and 458 patients from Memorial Sloan Kettering Cancer Center (MSKCC) were identified for use as a comparator cohort (Table 1). The median age of diagnosis was 55.8 years (range: 18.2–107.2) in the Nigerian cohort and 60.0 years (range: 18.1–97.4) in the MSKCC cohort (*P* < 0.001). There was a significant difference in the stage at presentation between the cohorts, with a higher proportion of Nigerian patients presenting with stage IV disease (53.8 vs. 36.2% MSKCC, *P* < 0.001). The location of the primary tumor differed significantly between the two cohorts, with more rectal primaries in Nigerian patients vs. MSKCC patients (50.8 vs. 33.7%, *P* < 0.001). In addition, MSKCC patients were more likely to present with lung (29.3 vs. 10.5%) and/or liver metastasis (41.0 vs. 26.8%) than patients in Nigeria (*P* < 0.001), whereas metastatic disease to the peritoneum was more common in the Nigerian cohort (30.3 vs. 18.1% MSKCC, *P* < 0.001).

**Table 1:** Sociodemographic and clinicopathologic data.

	MSKCC (*n* = 458)	Nigeria (*n* = 380)	*P* value^a^
Age of diagnosis, years			
Median (range)	60.0 (18.1–97.4)	55.8 (18.2–107.2)	<0.001
Sex			
Female	217 (47.4)	178 (46.8)	0.89
Male	241 (52.6)	202 (53.2)	
Diabetes	89 (19.4)	25 (6.6)	<0.001
Hypertension	237 (51.7)	82 (21.6)	<0.001
Family history of colorectal cancer	70 (15.3)	15 (3.9)	<0.001
Smoking history	216 (47.2)	41 (10.8)	<0.001
Unknown	2	0	
Stage^b^			
I	49 (10.7)	1 (0.3)	<0.001
II	79 (17.2)	41 (12.5)	
III	164 (35.8)	109 (33.3)	
IV	166 (36.2)	176 (53.8)	
Unknown	0	53	
Tumor location			
Cecum	52 (11.4)	9 (2.4)	<0.001
Right colon	69 (15.1)	82 (22.2)	
Transverse colon	25 (5.5)	18 (4.9)	
Left colon	33 (7.2)	18 (4.9)	
Sigmoid colon	124 (27.1)	55 (14.9)	
Rectum	154 (33.7)	188 (50.8)	
Unknown	1	10	
Location of metastases^c^			
Lung	134 (29.3)	40 (10.5)	<0.001
Liver	188 (41.0)	102 (26.8)	<0.001
Peritoneal	83 (18.1)	115 (30.3)	<0.001

Data are *n* (%) unless noted.

*MSKCC* Memorial Sloan Kettering Cancer Center.

^a^*P* values by Wilcoxon rank-sum test for continuous variables and Fisher’s exact test for categorical variables.

^b^For the MSKCC cohort, tumors treated with upfront surgery were staged pathologically and tumors treated with neoadjuvant therapy were staged clinically.

^c^Metastasis at presentation.

### Molecular comparison

A total of 157 Nigerian CRC tumor specimens underwent molecular profiling with a combination of IHC, next-generation sequencing (Memorial Sloan Kettering-Integrated Mutation Profiling of Actionable Cancer Targets (MSK-IMPACT)), and/or methylation analysis. A total of 64 Nigerian and 1145 MSKCC specimens underwent MSK-IMPACT analysis, including ancestry analysis for 64 of the Nigerian specimens and 604 of the MSKCC specimens. One hundred percent (64/64) of the Nigerian specimens were compatible with a genetically determined ancestry (GDA) of African origin. The MSKCC cohort was predominately of European GDA (466/604, 77.2%), followed by African (77/604, 12.7%), East Asian (37/604, 6.8%), South East Asian (17/604, 2.8%), and Native American GDA (7/604, 1.2%). Ninety-five percent (95.8%) of patients who self-reported as African American had a GDA of African origin. The ancestral analysis of the MSKCC and Nigerian cohorts is presented in Supplementary Fig. 1.

**Fig. 1:**
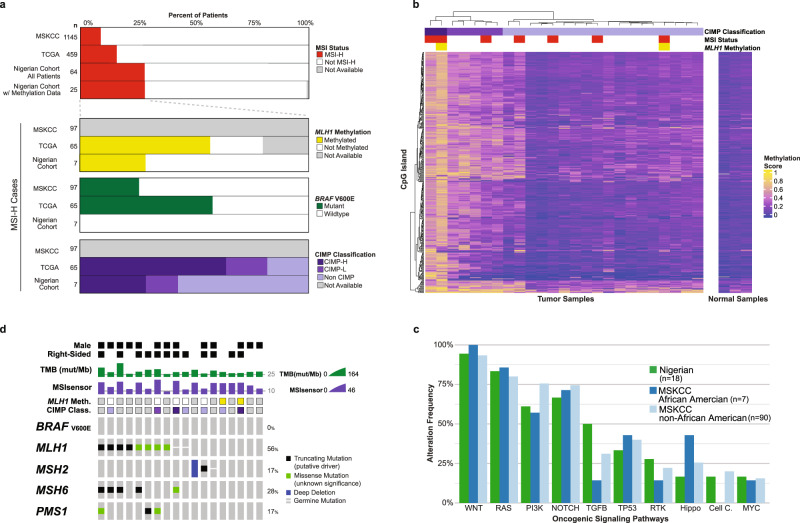
Molecular profile of MSI-H colorectal cancer patients. **a** Frequency of microsatellite instability high (MSI-H) by cohort (Nigerian, The Cancer Genome Atlas [TCGA], Memorial Sloan Kettering Cancer Center [MSKCC]). *MLH1* methylation, *BRAF* V600E mutation, and CpG island methylator phenotype (CIMP) frequencies are shown for MSI-H patients. **b** Methylation data from Nigerian patients are presented along with CIMP classification (i.e., high [CIMP-H] *n* = 2, low [CIMP-L] *n* = 5, non-CIMP *n* = 18) and MSI status (i.e., stable [MSS] *n* = 18, high [MSI-H] *n* = 7). **c** Frequency of oncogenic signaling pathway alterations in Nigerian and MSKCC MSI-H tumors (*n* = 18), with MSKCC patients stratified by African American and non-African American ethnicity. **d** Oncoprint of *BRAF* V600E and mismatch repair (MMR) genomic alterations (*MLH1*, *MSH2*, *MSH6*, *PMS1*) in MSI-H Nigerian specimens is presented with sex, location of primary, total mutational burden (TMB), methylation status, CIMP classification, and MSIsensor score.

Of the 64 Nigerian specimens that underwent MSK-IMPACT, 28.1% (18/64) were MSI-high (MSI-H), consistent with the rate of MMR protein deficiency by IHC (20/94, 21.3%). This was significantly higher than the rate of MSI-H in CRCs from The Cancer Genome Atlas (TCGA; 65/459, 14.2%) and MSKCC comparison cohorts (97/1145, 8.5%, *P* < 0.001, Fig. 1a). This trend persisted when the analysis was limited to patients ≤ 45 years of age (MSI-H 9.5% MSKCC vs. 18% Nigerian). The rate of MSI-H in the Nigerian cohort was also higher than the rate of MSI-H in the subset of MSKCC patients who self-identified as African American (28.1% vs. 7.2% [7/97]). MSI-H tumors were more common in the right colon (12/18, 66.7%) than the left colon (5/18, 27.8%) in Nigerian patients. MSIsensor score and total mutation burden (TMB) for Nigerian specimens stratified by MSI status are presented in Supplementary Fig. 2^11^. The phenotype of CRC, stratified by cohort and MSI status, is presented in Table 2. There was no difference in the median age between the cohorts (*P* = 0.57). Among rectal cancer patients, there was a significant difference in the MSI status between the Nigerian (65.2% microsatellite stable [MSS], 5.6% MSI-H) and MSKCC (25.1% MSS, 8.6% MSI-H) cohorts (*P* = 0.034). There was also a significant difference in the stage-at-presentation between cohorts, with a larger proportion of MSI-H patients presenting with stage IV disease in Nigeria vs. MSKCC (50.0 vs. 18.9%, *P* = 0.032). The median overall survival (OS) was not different between the cohorts by MSI status (*P* = 0.15).

**Table 2:** Clinicopathologic comparison by MSI status.

	MSKCC		Nigeria		*P* value^a^
	MSI-H (*n* = 106)	MSS (*n* = 997)	MSI-H (*n* = 18)	MSS (*n* = 46)	
*Median age at diagnosis, years (range)*	60.0 (20.0–85.0)	54.0 (13.0–93.0)	57.1 (31.4–84.6)	58.2 (27.7–83.3)	0.57
*Sex*					
Female	44 (41.5)	461 (46.2)	4 (22.2)	22 (47.8)	0.15
Male	62 (58.5)	536 (53.8)	14 (77.8)	24 (52.2)	
*Location*					
Left	23 (21.9)	462 (47.0)	5 (27.8)	8 (17.4)	REF
Rectum	9 (8.6)	247 (25.1)	1 (5.6)	30 (65.2)	0.03
Right	73 (69.5)	274 (27.9)	12 (66.7)	8 (17.4)	0.30
Unknown	1	14	0	0	
*Mucin*					
No	43 (56.6)	619 (86.5)	6 (33.3)	32 (72.7)	0.90
Yes	33 (43.4)	97 (13.5)	12 (66.7)	12 (27.3)	
Unknown	30	281	0	2	
*Stage*^b^					
I	7 (6.6)	34 (3.4)	0 (0)	0 (0)	0.03
II	40 (37.7)	91 (9.1)	3 (16.7)	5 (10.9)	
III	39 (36.8)	228 (22.9)	6 (33.3)	10 (21.7)	
IV	20 (18.9)	644 (64.6)	9 (50.0)	31 (67.4)	
*Location of metastatic disease*^c^					
Liver					
No	9 (45.0)	105 (16.3)	8 (88.9)	21 (67.7)	0.93
Yes	11 (55.0)	539 (83.7)	1 (11.1)	10 (32.3)	
Lung					
No	19 (95.0)	550 (85.4)	8 (88.9)	21 (67.7)	0.92
Yes	1 (5.0)	94 (14.6)	1 (11.1)	10 (32.3)	
Peritoneal					
No	14 (70.0)	539 (83.7)	4 (44.4)	18 (58.1)	0.79
Yes	6 (30.0)	105 (16.3)	5 (55.6)	13 (41.9)	
Other					
No	14 (70.0)	496 (77.0)	9 (100)	30 (96.8)	>0.95
Yes	6 (30.0)	148 (23.0)	0 (0)	1 (3.2)	
*Median overall survival, months (95% CI)*	NR (NR–NR)	69.1 (59.9–83.2)	32.1 (8.5–NR)	4.8 (2.1–10.6)	0.15

Data are *n* (%) unless noted.

*MSKCC* Memorial Sloan Kettering Cancer Center, *MSI-H* microsatellite instability high, *MSS* microsatellite stable, *CI* confidence interval, *NR* not reported.

^a^The covariates included the main effects molecular subtype and center and an interaction term of the two. A linear model was used for age, a logistic model for gender, mucin, stage, location of metastatic disease, a multinomial model for location of primary, and a Cox model for overall survival. *P* values by Wilcoxon rank-sum test for continuous variables, Fisher’s exact test for categorical variables, and log rank test for overall survival.

^b^The comparison is stage I, II, III vs. IV.

^c^In the subset of patients presenting with stage IV.

Methylation analysis was used to further characterize the molecular pathogenesis of the abnormally high incidence of MSI-H specimens. Convenience sampling was used to select a subset of the specimens examined with MSK-IMPACT (25/64). Hierarchical clustering (Euclidian distance, complete linkage clustering) comparing the 95th percentile of variant genomic windows revealed that 32.0% (8/25) of specimens were highly methylated. Overall, 7/25 (28.0%) of specimens were MSI-H, and *MLH1* was highly methylated in 2/7 (28.6%) of the MSI-H cases (Fig. 1a). Two of the 25 (8.0%) specimens that underwent methylation analysis were CpG island methylator phenotype-high (CIMP-H) (Fig. 1b).

We also examined the frequency of somatic oncogenic alterations between Nigerian (*n* = 18) and MSKCC (*n* = 97) MSI-H patients. The *BRAF* V600E mutation was not present in any of MSI-H Nigerian specimens (0/18) compared to 25.8% (25/97) in the MSKCC cohort (*P* = 0.01). *KRAS* mutations were more common in Nigerian compared to MSKCC MSI-H patients, although the difference was not statistically significant (61.1 vs. 41.1%, *P* = 0.20). In the Nigerian cohort, 58.3% (7/12) of right-sided vs. 66.7% (4/6) of left-sided tumors harbored a *KRAS* mutation. In both cohorts, *APC* was altered at a similar frequency (55.6 vs. 52.2%, *P* = 1.0). *APC* mutations were present in 50% (6/12) of right-sided tumors vs. 66.7% (4/6) of left-sided tumors in the Nigerian cohort compared to 39.3% (24/61) and 78.6% (22/28) of non-African American (non-AA) patients at MSKCC. *TCF7L2* was more frequently altered in Nigerian patients (64.3 vs. 34.2% MSKCC, *P* = 0.04). There were no differences in the frequency of alterations in ten commonly altered oncogenic signaling pathways involved in CRC tumorigenesis between the Nigerian and MSKCC MSI-H cohorts, including MSKCC patients who self-identified as African American (Fig. 1c).

The high frequency of MSI-H tumors and the absence of activating mutations in *BRAF* in the Nigerian CRC specimens was suggestive of germline etiology. Anonymous germline mutation analysis was performed on the Nigerian patients with MSI-H tumors (*n* = 18) using the MSK-IMPACT data (Table 3). A high-penetrance pathogenic or likely pathogenic germline variant was identified in 22.2% (4/18) of the patients. Three of 18 (16.7%) patients had germline mutations in DNA MMR genes (two in *MLH1*, one in *MSH2*), consistent with a diagnosis of Lynch Syndrome (Fig. 1d). The fourth patient had an incidental finding of a *BRCA1* mutation. In the 46 MSS tumors, a single pathogenic *APC* mutation, consistent with a diagnosis of familial adenomatous polyposis, as well as another pathogenic mutation in *MLH1* were identified.

**Table 3:** Germline mutations in Nigerian patients with colorectal cancer.

MSI status	*n*	*MLH1*	*MSH2*	*APC*	*BRCA1*	Total germline mutations	Total MMR gene mutations
MSI-H	18	2	1	0	1	4 (22.2%)	3 (16.7%)
MSS	46	1	0	1	0	2 (4.3%)	1 (2.2%)
Total	64	3	1	1	1	6 (9.3%)	4 (6.3%)

*MSI* microsatellite stable, *MSI-H* microsatellite instability high, *MSS* microsatellite stable, *MMR* mistmatch repair.

**Fig. 2:**
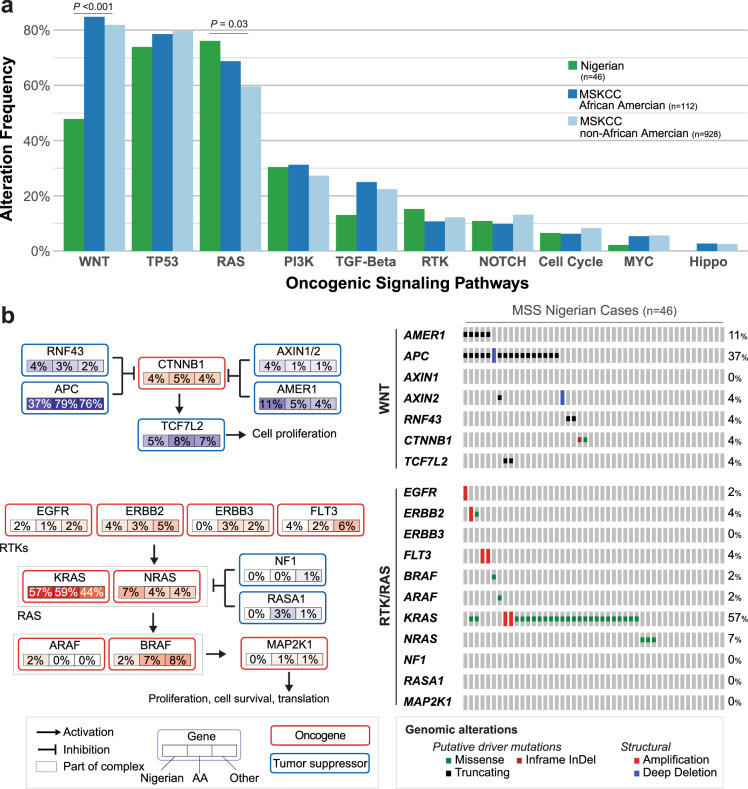
Molecular profile of MSS colorectal cancer patients. **a** Frequency of oncogenic signaling pathway alterations in Nigerian and Memorial Sloan Kettering Cancer Center (MSKCC) patients, with MSKCC patients stratified by African American and non-African American ethnicity. **b** Specific genomic alterations and frequencies within the WNT and RAS oncogenic pathways for the Nigerian microsatellite stable (MSS) patients (*n* = 46). AA African American, InDel insertion or deletion. *P* values by two-sided Fisher’s exact test.

In MSS specimens (Nigerian *n* = 46, MSKCC *n* = 1040), *TP53* (71.7% Nigeria vs. 77.5% MSKCC, *P* = 0.370) and *KRAS* (56.5% Nigeria vs. 44.1% MSKCC, *P* = 0.13) somatic mutation frequencies were not different between the cohorts. *TP53* was less frequently altered in right-sided tumors in the Nigerian cohort (25 vs. 81.6% left-sided) compared to non-AA patients as MSKCC (66.7 vs. 81.4% left-sided). *KRAS* was altered in 62.5% (5/8) of right-sided tumors and 55.3% (21/38) of left-sided tumors in the Nigerian cohort compared to 64% (146/228) and 38.2% (256/688), respectively, at MSKCC (non-AA). *APC* was less likely to be altered in the Nigerian MSS cohort (36.9 vs. 76.0% MSKCC, *P* < 0.01). *APC* was altered in 25% (2/8) of right-sided and 42.1% (16/38) of left-sided Nigerian patients compared to 68.4% (156/228) and 78.8% (542/688), respectively, at MSKCC (non-AA). Despite the lower frequency of *APC* alterations in the Nigerian MSS cohort, *CTNNB1* gene alterations were similar between the two groups (4.4 vs. 3.7%, *P* = 0.68). Nigerian patients were also more likely than MSKCC patients to have PI3K pathway alterations, including *PIK3R1* (8.7 vs. 2.2%, *P* = 0.02) and *PIK3CB* (4.3 vs. 0.5%, *P* = 0.04). Mutations in the MSS tumors were further grouped by canonical signaling pathways (Fig. 2a). Nigerian patients had a lower frequency of WNT pathway alterations (47.8 vs. 81.9% MSKCC, *P* < 0.001). The Nigerian MSS cohort also had a higher frequency of RAS pathway alterations as compared to MSKCC MSS patients of non-AA ethnicity (76.1 vs. 59.6%, *P* = 0.03). Alterations within the WNT and RAS pathways in the Nigerian MSS specimens are presented in Fig. 2b.

### Treatment and outcomes comparison

Given the differences in presentation and molecular profile detected, we compared treatment modalities and outcomes between the Nigerian and MSKCC cohorts (Supplementary Table 1). A larger number of MSKCC patients received neoadjuvant chemotherapy (83.8%) compared to Nigerian patients (57.5%, *P* < 0.001). A larger number of MSKCC patients also underwent surgery (87.3%) during their treatment compared to Nigerian patients (71.0%, *P* < 0.001).

The median follow-up at MSKCC was 64.8 months and 16.9 months for the Nigerian cohort. The median OS for the MSKCC cohort has not yet been reached (95% confidence interval (CI): 71 months–NR) compared to 11.9 months (95% CI: 10.2–14.5) for the Nigerian cohort (*P* < 0.001). Median OS for rectal (NR) and colon cancer (71.1 months, 95% CI: 59.4 months–NR) patients were significantly longer at MSKCC than for both cohorts of patients in Nigeria (colon: 13.3 months, 95% CI: 9.5–17.9 months; rectal: 11.0 months, 95% CI: 9.2–14.2 months; *P* < 0.001) (Supplementary Fig. 3a, b). OS was significantly longer for patients presenting with stage IV disease at MSKCC (34.3 months, 95% CI: 28.6–41.8) compared to Nigeria (7.3 months, 95% CI: 5.3–9.4, *P* < 0.001). Patients at MSKCC also had significantly longer recurrence-free survival (60.4 MSKCC vs. 12.5 months Nigeria, *P* < 0.001, Supplementary Fig. 3c). Notably, in the Nigerian cohort, the majority of the events in this analysis were death (i.e., death of disease) as opposed to recurrence.

## Discussion

The burden of CRC is increasing in Nigeria^12^, and the Nigerian Federal Ministry of Health’s National Cancer Control Plan (NCCP) identifies strategies to improve the early detection and treatment of CRC as a priority. Although literature from the US suggests a unique profile of CRC among African Americans, there is a glaring dearth of prospective data from sub-Saharan Africa^16^. We present a comprehensive evaluation of the molecular profile and phenotype of CRC in Nigeria as a critical step toward addressing the NCCP recommendations. Our results suggest a younger age of disease onset, a higher burden of rectal and stage IV disease, and worse stage-for-stage OS in Nigerian CRC patients as compared to CRC patients in the US^5^. Nigerian patients, who were exclusively of African GDA (100%), were more than twice as likely to have MSI-H CRC than patients from MSKCC, who were predominately of European GDA (77.2%). This is consistent with previous data from the region^9,10,13,14^.

In Nigerian patients, germline analysis revealed that only 17% of MSI-H specimens had a pathologic MMR gene germline mutation, similar to the rate expected in MSI-H patients in HICs (11.0–18.5%)^17,18^. Typically, epigenetic methylation of the promoter region of *MLH1* accounts for the majority of somatic MSI-H CRC^10,19^. In our methylation analysis, less than a third of MSI-H Nigerian CRC specimens demonstrated *MLH1* methylation, and only 8% were CIMP-H. These findings suggest that an alternative molecular pathway, such as biallelic somatic inactivation, may play a dominant role in somatic MSI-H CRC tumorigenesis in Nigerian patients^20^. This represents a departure from the dominant pathway of MSI tumorigenesis in HICs.

The molecular profile of sporadic MSS CRC in HICs is characterized by a high frequency of inactivating *APC* and/or activating *CTNNB1* mutations (>80%), which drive cell proliferation through the WNT pathway^21,22^. Among MSS CRCs from Nigerian patients, there were significantly fewer somatic *APC* mutations vs. MSKCC patients (37 vs. 76%, *P* < 0.01). This difference persisted when alteration frequency was examined by sidedness of the primary tumor. The low frequency of WNT pathway alterations among Nigerian MSS patients suggests that this pathway is not the dominant driver of MSS tumorigenesis in this population.

The differences in the molecular profile of CRC raise questions regarding disease management in Nigeria. The greater burden of MSI-H CRC in Nigeria should be considered in recommendations for routine MSI/MMR testing and treatment guidelines for systemic therapy^23^. Based on our data, it is possible that up to a quarter of Nigerian CRC patients (i.e., those with MSI-H tumors) would derive marginal benefit from systemic fluorouracil-based chemotherapy. Limiting the use of chemotherapy in early-stage disease and accelerating the use of immunotherapy for MSI-H tumors requires examination and may improve outcomes. The mutational profile of CRC in Nigeria also suggests that caution should be taken before generalizing therapeutic trial results produced in the US. This insight may have implications for other LMICs as well, as international resource-stratified screening and treatment guidelines for CRC are being proposed and adopted^24^.

This study has several limitations. Staging was not concordant between the MSKCC and Nigeria cohorts, due to a lack of pathologic staging for many Nigerian patients who never underwent resection. As an exploratory analysis, the number of Nigerian specimens available for molecular comparison was also small and limits the strength of our conclusions and our ability to conduct certain analyses (e.g., somatic alteration by ancestry, statistical comparison by sidedness of tumor). Tissue availability for the methylation analysis was particularly limited, and the results of this analysis should be interpreted with caution. This limited our ability to construct a suitable internal validation cohort and an external validation cohort does not currently exist in the public domain. Thus, our findings are hypothesis-generating and lay the foundation for larger studies with broader geographic and sociodemographic coverage. The use of the MSKCC and TCGA cohorts as comparators may also introduce an element of selection bias. MSKCC is a specialized cancer center with a unique patient population that may not be generalizable to the US as a whole, and the TCGA cohort is predominantly of European descent.

In summary, the molecular profile and phenotype of CRC in Nigeria may differ from that in HICs. The greater burden of MSI-H CRCs in Nigeria needs to be examined in larger cohorts but should be considered in recommendations for routine MSI/MMR testing. Our data have implications for resource-stratified screening and treatment guidelines for CRC and emphasize the need for regional data to guide diagnostic and treatment approaches for patients in LMICs.

## Methods

### Study design and data collection

We evaluated prospectively collected CRC data and specimens obtained through the African Research Group for Oncology (ARGO), a partnership between MSKCC, Obafemi Awolowo University (OAU), and 12 tertiary care facilities across Nigeria. A prospectively maintained REDCap (Research Electronic Data Capture, Vanderbilt University) database has been used to collect demographic and outcome data for patients with CRC presenting at Nigerian ARGO sites since 2013. Fresh-frozen tumor and blood specimens from each participant are obtained for a prospectively maintained biobank. Outcome data are collected at the time of routine follow-up.

All patients enrolled in the ARGO CRC database from April 2013 to November 2018 were included in the present analysis. Patients aged <18 years and those without histologically confirmed colorectal adenocarcinoma were excluded. For clinicopathological comparisons, data from MSKCC were extracted from a prospective database and from the electronic medical record using ICD-O histological diagnosis consistent with CRC. All new patients aged >18 years with colorectal adenocarcinoma diagnosed from January 2013 to June 2013 were included in the MSKCC cohort.

For both cohorts, information was collected on patient sociodemographics, clinical presentation, histopathology, and outcomes as of the last date of follow-up. For the MSKCC cohort, the date of diagnosis was defined as the date of pathologic diagnosis. Clinical staging was used for patients who received neoadjuvant therapy, and pathological staging was used for patients treated with upfront surgery, as previously reported^25^. For the Nigerian cohort, the date of diagnosis was defined as the date of clinical diagnosis, and all patients were staged clinically. Metastatic disease at diagnosis was defined as metastases documented on initial staging or metastases found intraoperatively. Primary tumor site was designated as right-sided for tumors from the cecum to the distal transverse colon and left-sided for tumors from the distal transverse colon/splenic flexure to the rectum. Date of diagnosis of metachronous metastasis was the date of first radiographic or biopsy-proven evidence of metastasis.

### Molecular profiling

The first half of consecutive Nigerian patients from the ARGO database used in the clinicopathologic evaluation underwent anonymized molecular profiling. For these patients, fresh-frozen tumor and matched venous blood samples were sent from Nigeria to MSKCC for paired tumor and germline genomic profiling. Targeted next-generation sequencing analysis was performed using the MSK-IMPACT assay, as previously described^26,27^. Somatic alterations, including single-nucleotide variants, small insertions/deletions, and copy number alterations, were identified. Germline variant calling used a modified version of a sequence analysis pipeline validated for CLIA-certified clinical use^27,28^. Pathogenicity of germline variants were classified based on American College of Medical Genetics and Genomics criteria^29^. Somatic genomic alterations were filtered for oncogenic variants using the OncoKB knowledgebase^30^. The total number of non-synonymous mutations was quantified for each sample and divided by the number of bases analyzed to calculate TMB. ADMIXTURE v1.3 was used to estimate the ancestry of each patient based on the five references populations utilized in the 1000 Genomes Project: African, European, East Asian, Native American, and South Asian. As previously described, GDA was based on the highest proportion reference population for each patient^31^.

MSK-IMPACT data were also used for MSIsensor analysis, a prospectively validated algorithm for assessing MSI/MMR status^32,33^. IHC was used to supplement MSIsensor assessment due to cost and resource limitations, which precluded next-generation sequencing of the entire cohort. Primary monoclonal antibodies against MLH1 (clone G168-728, diluted 1:250, BD PharMingen, San Diego, CA), MSH2 (clone FE11, diluted 1:50, Oncogene Research Products, La Jolla, CA), MSH6 (clone 44, ready to use, Ventana Medical Systems Inc., Oro Valley, AZ), and PMS2 (clone A16-4, diluted 1:200, BD PharMingen) were used. Finally, a subset of the samples underwent genome-wide DNA methylation analysis by reduced representation bisulfite sequencing using the Zymo Methyl-MiniSeq platform (Zymo Research, Irvine, CA).

Data from the Nigerian cohort were compared to a cohort of CRC specimens processed by the Molecular Diagnostics Service at MSKCC between April 2014 and September 2016. Comparison was also made to data from CRC specimens included in TCGA obtained from http://cancergenome.nih.gov/.

### Immunohistochemistry

IHC was performed on 4-μm-thick sections of the fresh-frozen specimens with a BenchMark XT automated immunostainer (Ventana Medical Systems Inc., Tucson, AZ). Primary monoclonal antibodies against MLH1 (clone G168-728, diluted 1:250, BD PharMingen, San Diego, CA), MSH2 (clone FE11, diluted 1:50, Oncogene Research Products, La Jolla, CA), MSH6 (clone 44, ready to use, Ventana Medical Systems Inc.), and PMS2 (clone A16-4, diluted 1:200, BD PharMingen) were used. For external controls, non-neoplastic colonic mucosa and colorectal tumors known to be deficient in MLH1, MSH2, MSH6, and PMS2 were employed. Evidence of expression of each protein was defined by nuclear IHC reactivity. The absence of MMR protein expression was defined by the total absence of nuclear staining. Even very weak staining was considered proficient.

### Methylation analysis

The raw BED files were filtered to remove all reads mapped to chromosomes other than 1–22 and converted to methylation call files formatted for analysis using R/Bioconductor package, “methylKit” version 0.99.2. Methylation call files were filtered by coverage to exclude loci with <10 reads, as well as the 99.9th percentile of each sample’s reads to reduce PCR bias. Using loci targeted by Infinium’s HumanMethylation450 BeadChip’s probes as a guide, we computed percent methylation for genomic windows 51 bp long (25 bp upstream/downstream from each probe’s target) and excluded windows exhibiting methylation >70%.

### Ancestry analysis

We identified autosomal biallelic single-nucleotide polymorphisms (SNPs) with minor allele frequency >1% in the 1000 Genomes Project and captured by MSK-IMPACT. We genotyped these sites in all consented MSKCC and Nigerian patients using GATK v4.0 HaplotypeCaller and Genotype GVCFs and merged with the 1000 Genomes data using PLINK v1.9. We then performed linkage-disequilibrium pruning and filtered sites with missing call rates of <0.1 using PLINK, which resulted in 4263 SNP markers. Samples from the 1000 Genomes Project were used to establish a reference set composed of five populations: African, European, East Asian, Native American, and South Asian. We ran supervised analysis in ADMIXTURE v1.3 to estimate ancestral proportions for the specimens.

### Statistical analysis

Descriptive statistics, median and range, were used for continuous variables, with comparisons made using Wilcoxon rank-sum test. Count and percentage were used for categorical variables, with comparisons made using Fisher’s exact test. Kaplan–Meier methods and log rank test were used to assess OS and recurrence-free survival. OS was defined as the time from diagnosis to death. Recurrence-free survival analysis included only patients who underwent surgery and was defined as time from surgery to recurrence or death.

To test for associations between variables of interest and molecular subtype, various interaction models were employed. The covariates included in these interaction models were the main effects molecular subtype and study cohort (e.g., MSKCC). To compare the frequency of oncogenic alterations between groups, two-sided Fisher’s exact tests were performed. Pathway analysis was conducted utilizing the pathway templates from Sanchez-Vega et al.^34^. The results of the genomic analyses were considered hypothesis-generating and were not corrected for multiple hypothesis testing due to the small sample size. SAS version 9.4 (SAS Institute Inc., Cary, NC) was used for all analyses. All tests were two-sided, with *P* < 0.05 considered significant.

### Ethical approval

Ethical approval for enrolment and maintenance of the ARGO prospective database was granted by the OAU Institutional Review Board (IRB). All patients provided written informed consent for tissue and data collection. Separate ethical clearance was granted to conduct anonymized molecular profiling, including germline mutation analysis, by the OAU IRB. Approval was also obtained from the MSKCC IRB (IRB# 15–209) for use of previously captured patient data for the clinicopathologic and molecular profile comparisons; all patients provided written informed consent for molecular analysis of tissue at MSKCC via protocols approved by both the OAU and MSKCC IRBs. All tissue and blood handling protocols and transcontinental transfer details were approved by the OAU IRB as the IRB of record.

### Reporting summary

Further information on research design is available in the Nature Research Reporting Summary linked to this article.

## Supplementary information

Supplementary Information

Reporting Summary

## Data Availability

The next-generation genomic sequencing data, including somatic mutation frequency and MSIsensor output, have been deposited in cBioPortal (www.cbioportal.org/study/summary?id=crc_nigerian_2020). The TCGA publicly available data used in this study are available in the GDC Data Portal under accession phs000178. The clinical data, including patient demographics, histopathology, treatment, and outcomes, are stored on a REDCap database at MSKCC. The clinical data are protected and are not available due to data privacy laws. This data may be available from the corresponding author after completion of data transfer agreements and research ethics board approval between all relevant parties. The remaining data are available within the article, Supplementary Information, or Source Data files. Source data are provided with this paper.

## Source data

Source Data
