# Supplementary material for: Molecular and phenotypic profiling of colorectal cancer patients in West Africa reveals biological insights
Source: Nat Commun. 2021 Nov 24;12:6821. doi: 10.1038/s41467-021-27106-w (PMC8613248; doi:10.1038/s41467-021-27106-w)
Supplement: Supplementary file 1 — Supplementary Information [file 41467_2021_27106_MOESM1_ESM.pdf]

SUPPLEMENTARY INFORMATION

Supplementary Figure 1: Proportional representation of genetically determined ancestry for patients from Memorial Sloan Kettering Cancer Center (MSKCC) and Nigeria (Nigerian *n* = 63, MSKCC African American *n* = 65, MSKCC Other Races *n* = 539). EUR: European, EAS: East Asian, NAM: Native American, SAS: South East Asian, AFR: African.

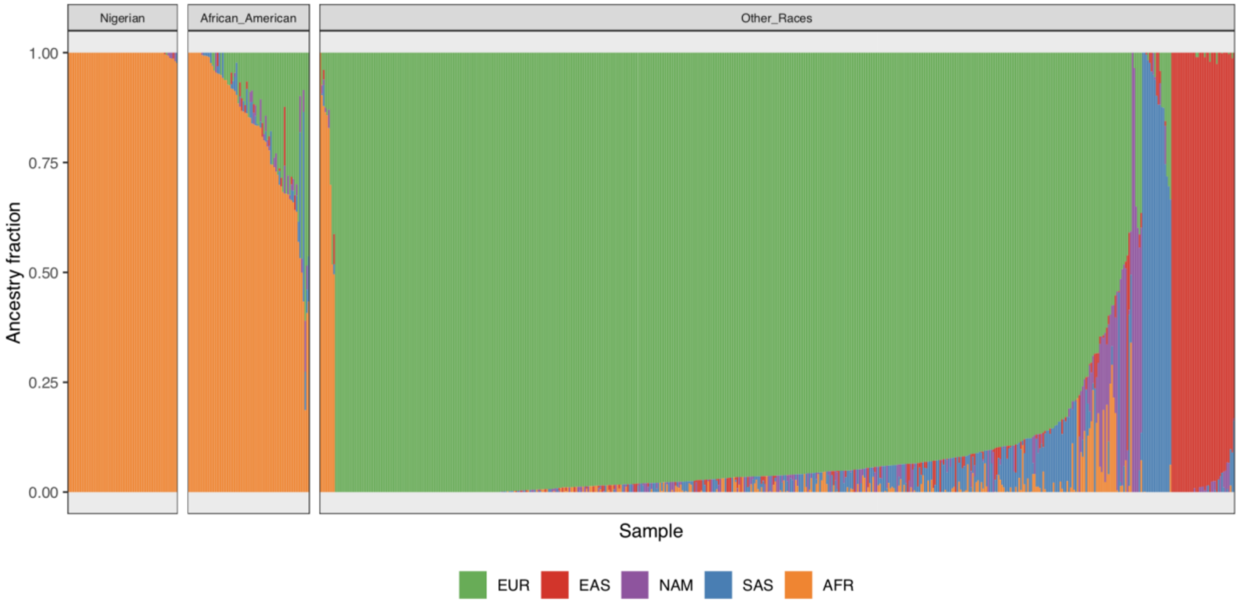

Supplementary Figure 2: MSIsensor score (**a**) and total tumor mutation burden (**b**) by molecular subtype (MSI status) for Nigerian colorectal cancer specimens (*n* = 64). *P* value by 2-sided Wilcoxon sum test. Boxplots show distribution with the minimum, maximum, median (line), and the first and third quartiles (bottom and top of box, respectively). MSI-H: microsatellite instability high, MSS: microsatellite stable.

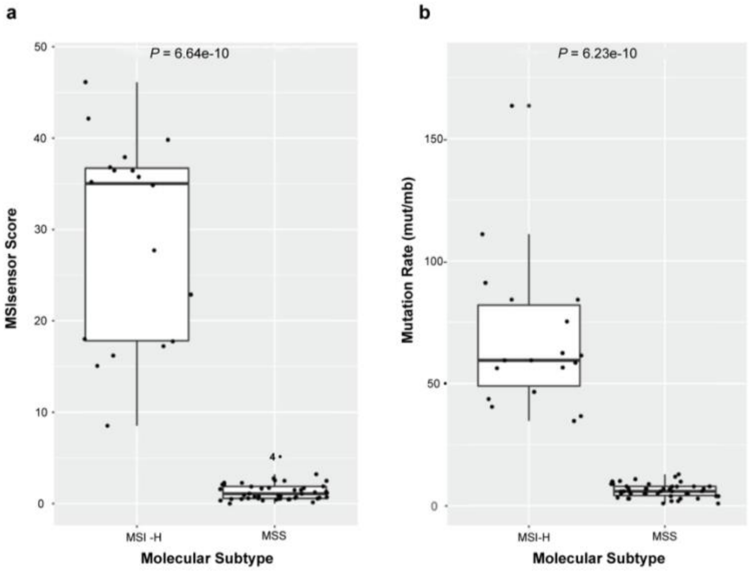

Supplementary Table 1: Treatment comparison

**Treatment Modality**	**MSKCC (*n* = 458)**	**Nigeria (*n* = 380)**	***P* value***
Surgery	400 (87.3)	247 (71.0)	<0.001
Unknown	0	32	
Chemotherapy	321 (70.1)	177 (61.0)	0.01
Unknown	0	90	
Timing of chemotherapy			
Adjuvant	52 (16.2)	74 (42.5)	<0.001
Neoadjuvant	269 (83.8)	100 (57.5)	
Unknown	0	3	
Pelvic radiotherapy (rectal cancer only)			
Yes	98 (63.6)	9 (4.8)	<0.001
No	56 (36.4)	179 (95.2)	
Surgery for stage IV disease	106 (71.6)	124 (67.0)	0.40
Unknown	0	2	

* *P* values determined by two-sided Fisher's exact test

Supplementary Figure 3: (**a**) Overall survival, (**b**) overall survival stratified by colon and rectal primary, and (**c**) recurrence-free survival for Memorial Sloan Kettering (MSK) Cancer Center vs. Nigerian cohorts.

a

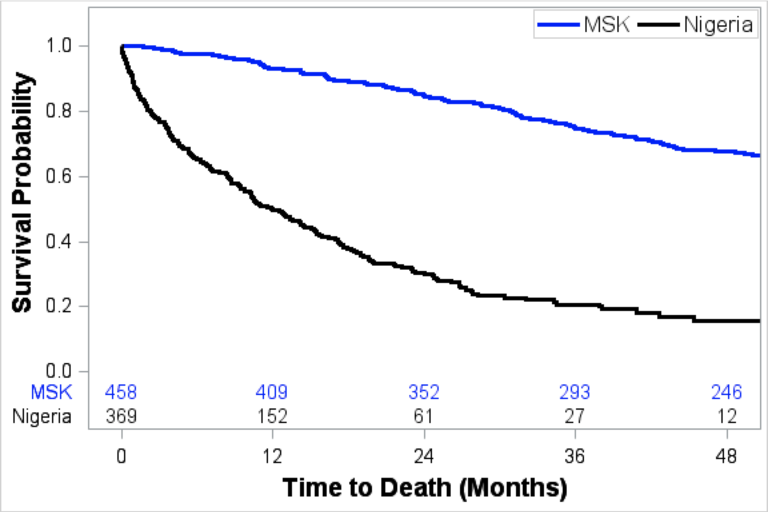

b

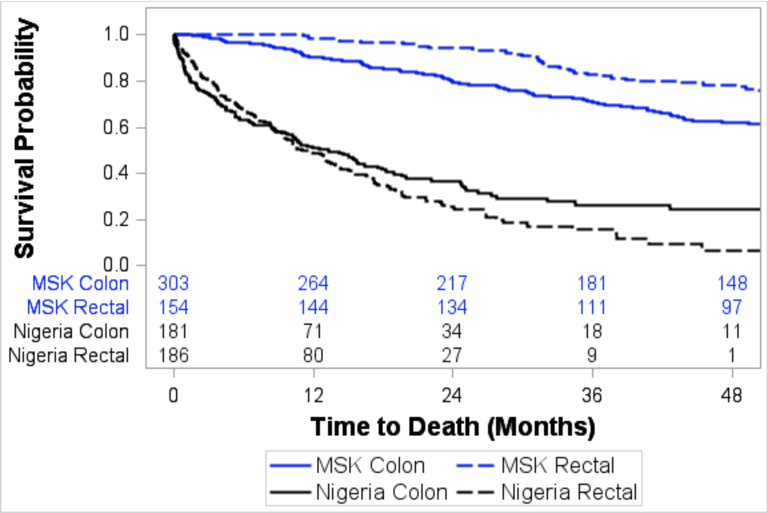

c

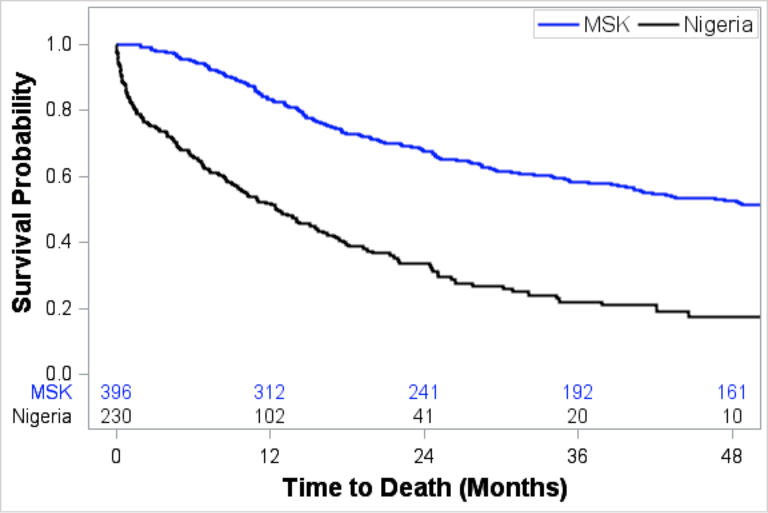
MSK
Nigeria
1.0
0.8
0.6
0.4
0.2
0.0
Survival Probability
MSK 396 312 241 192 161
Nigeria 230 102 41 20 10
0
12
24
36
48
Time to Death (Months)
