# Supplementary material for: Molecular and phenotypic profiling of colorectal cancer patients in West Africa reveals biological insights
Source: Nat Commun. 2021 Nov 24;12:6821. doi: 10.1038/s41467-021-27106-w (PMC8613248; doi:10.1038/s41467-021-27106-w)
Supplement: Supplementary file 2 — Reporting Summary [file 41467_2021_27106_MOESM2_ESM.pdf]

nature research

Corresponding author(s): T. Peter Kingham

Last updated by author(s): October 25, 2021

Reporting Summary

Nature Research wishes to improve the reproducibility of the work that we publish. This form provides structure for consistency and transparency in reporting. For further information on Nature Research policies, see our Editorial Policies and the Editorial Policy Checklist.

Statistics

For all statistical analyses, confirm that the following items are present in the figure legend, table legend, main text, or Methods section.

n/a	Confirmed	
☐	☒	The exact sample size (*n*) for each experimental group/condition, given as a discrete number and unit of measurement
☒	☐	A statement on whether measurements were taken from distinct samples or whether the same sample was measured repeatedly
☐	☒	The statistical test(s) used AND whether they are one- or two-sided<br>*Only common tests should be described solely by name; describe more complex techniques in the Methods section.*
☐	☒	A description of all covariates tested
☒	☐	A description of any assumptions or corrections, such as tests of normality and adjustment for multiple comparisons
☐	☒	A full description of the statistical parameters including central tendency (e.g. means) or other basic estimates (e.g. regression coefficient) AND variation (e.g. standard deviation) or associated estimates of uncertainty (e.g. confidence intervals)
☐	☒	For null hypothesis testing, the test statistic (e.g. *F*, *t*, *r*) with confidence intervals, effect sizes, degrees of freedom and *P* value noted<br>*Give P values as exact values whenever suitable.*
☒	☐	For Bayesian analysis, information on the choice of priors and Markov chain Monte Carlo settings
☒	☐	For hierarchical and complex designs, identification of the appropriate level for tests and full reporting of outcomes
☒	☐	Estimates of effect sizes (e.g. Cohen's *d*, Pearson's *r*), indicating how they were calculated

Our web collection on statistics for biologists contains articles on many of the points above.

Software and code

Policy information about availability of computer code

Data collection	No software was used for data collection
Data analysis	R platform v3.6.1, only trivial code used SAS version 9.4, ADMIXTURE v1.3

For manuscripts utilizing custom algorithms or software that are central to the research but not yet described in published literature, software must be made available to editors and reviewers. We strongly encourage code deposition in a community repository (e.g. GitHub). See the Nature Research guidelines for submitting code & software for further information.

Data

Policy information about availability of data

All manuscripts must include a data availability statement. This statement should provide the following information, where applicable:

- Accession codes, unique identifiers, or web links for publicly available datasets
- A list of figures that have associated raw data
- A description of any restrictions on data availability

The next-generation genomic sequencing data, including somatic mutation frequency and MSIsensor output, have been deposited in cBioPortal (www.cbioportal.org/study/summary?id=crc_nigerian_2020). The TCGA publicly available data used in this study are available in the GDC Data Portal under accession phs000178 (https://www.ncbi.nlm.nih.gov/projects/gap/cgi-bin/study.cgi?study_id=phs000178.v11.p8). The clinical data, including patient demographics, histopathology, treatment and outcomes are stored on a REDCap database at MSKCC. The clinical data are protected and are not available due to data privacy laws. This data may be available from the corresponding author after completion of data transfer agreements and research ethics board approval between all relevant parties. The remaining data are available within the Article, Supplementary Information or Source Data files

Field-specific reporting

Please select the one below that is the best fit for your research. If you are not sure, read the appropriate sections before making your selection.

- [x] Life sciences
- [ ] Behavioural & social sciences
- [ ] Ecological, evolutionary & environmental sciences

For a reference copy of the document with all sections, see nature.com/documents/nr-reporting-summary-flat.pdf

Life sciences study design

All studies must disclose on these points even when the disclosure is negative.

Sample size	Sample were taken from a prospective collected biobank and represent consecutive patients treated at the principle facility. Sample size was determined by the number of available samples and the unique resource and logistical constraints of next-generation sequencing samples from rural Nigeria.
Data exclusions	Only one specimen from Nigeria cohort was excluded from the interpretation of the next-generation sequencing results due to insufficient tissue.
Replication	For the central finding of the manuscript which is the higher-incidence of microsatellite instability, this was replicated twice. The finding was demonstrated using both next-generation sequencing and immunohistochemistry. The very small sample size precluded external validation and a suitable validation cohort does not currently exist in the literature.
Randomization	Allocation was not random. This was a cross-sectional study design of consecutive patients collected prospectively from a single institution in rural Nigeria. Given the small sample size we did not have sufficient power to control for co-variants such as age or sex, but present the raw data and explicitly address this weakness in the manuscript.
Blinding	As a descriptive cross-sectional analysis of the molecular profile of consecutive patients in Nigeria, blinding was not performed.

Reporting for specific materials, systems and methods

We require information from authors about some types of materials, experimental systems and methods used in many studies. Here, indicate whether each material, system or method listed is relevant to your study. If you are not sure if a list item applies to your research, read the appropriate section before selecting a response.

Materials & experimental systems

n/a	Involved in the study	
☐	☒	Antibodies
☒	☐	Eukaryotic cell lines
☒	☐	Palaeontology and archaeology
☒	☐	Animals and other organisms
☐	☒	Human research participants
☒	☐	Clinical data
☒	☐	Dual use research of concern

Methods

n/a	Involved in the study	
☒	☐	ChIP-seq
☒	☐	Flow cytometry
☒	☐	MRI-based neuroimaging

Antibodies

Antibodies used	MLH1 (clone G168-728, diluted 1:250, BD PharMingen, San Diego, CA)<br>MSH2 (clone FE11, diluted 1:50, Oncogene Research Products, La Jolla, CA)<br>MSH6 (clone 44, ready to use, Ventana Medical Systems Inc.)<br>PMS2 (clone A16-4, diluted 1:200, BD PharMingen)
Validation	MLH1 (clone G168-728): https://www.bdbiosciences.com/us/applications/research/apoptosis/purified-antibodies/purified-mouse-anti-human-mlh1-g168-728/p/554073<br>MSH2 (clone FE11): https://www.emdmillipore.com/US/en/product/Anti-MSH2-Antibody-clone-FE11,MM_NF-MABE284<br>MSH6 (clone 44): https://diagnostics.roche.com/ir/en/products/tests/confirm-anti-msh6-44-mouse-monoclonal-primary-antibody.html<br>PMS2 (clone A16-4) https://www.bdbiosciences.com/us/applications/research/apoptosis/purified-antibodies/purified-mouse-anti-pms2-a16-4/p/556415

Human research participants

Policy information about studies involving human research participants

Population characteristics

We evaluated prospectively collected CRC data and specimens obtained through the African Research Group for Oncology (ARGO), a partnership between Memorial Sloan Kettering Cancer Center (MSKCC), Obafemi Awolowo University (OAU), and 12 tertiary care facilities across Nigeria. All adult patients (<18 years of age) enrolled in the ARGO CRC database from April 2013-November 2018 were included in the present analysis. All new patients >18 years with colorectal adenocarcinoma diagnosed from January 2013-June 2013 were included in the MSKCC cohort.Those without histologically confirmed colorectal adenocarcinoma were excluded. At MSKCC, the median patient age was 60.0 years vs. 55.8 within the ARGO (Nigeria) database. At MSKCC, 52.6% of patients were male vs. 53.2% in Nigeria. Only 3.9% of Nigerian patients reported a family history of CRC vs. 15.3% at MSKCC.

Recruitment

Since 2013, ARGO has been prospectively capturing demographic, surgical, pathologic, radiologic, and outcomes data on adult patients who present with CRC at ARGO centers in Nigeria. Subjects are identified by a member of the patient's treatment team, the site investigator, or research team at OAU. Prior to study entry, the study staff explains to each potential subject the research objectives, risks and benefits of participation, and the subject's rights and responsibilities. If the patient agrees to participate, informed consent is obtained by a member of the study team. Participation is completely voluntary. Demographic, presentation, and staging data is obtained from the medical record; tissue from primary tumors is collected by colonoscopy or, if there is a T4 tumor, by biopsy during laparotomy; a peripheral blood sample is also collected. Biological samples are stored in the ARGO biobank. Both OAU and MSKCC are specialized referral centers for CRC care. The patients presenting at both institutions may not reflect the average CRC patient in the community setting or either country as a whole.

Ethics oversight

Ethical approval for enrolment and maintenance of the ARGO prospective database was granted by the OAU Institutional Review Board (IRB). All patients provided written informed consent for tissue and data collection. Separate ethical clearance was granted to conduct anonymized molecular profiling, including germline mutation analysis, by the OAU IRB. Approval was also obtained from the MSKCC IRB (IRB# 15-209) for use of previously captured patient data for the clinicopathologic and molecular profile comparisons; all patients provided consent for molecular analysis of tissue at MSKCC via protocols approved by both the OAU and MSKCC IRBs. All tissue and blood handling protocols and transcontinental transfer details were approved by the OAU IRB as the IRB of record.
